# Relevant Obstetric Factors for Cerebral Palsy: From the Nationwide Obstetric Compensation System in Japan

**DOI:** 10.1371/journal.pone.0148122

**Published:** 2016-01-28

**Authors:** Junichi Hasegawa, Satoshi Toyokawa, Tsuyomu Ikenoue, Yuri Asano, Shoji Satoh, Tomoaki Ikeda, Kiyotake Ichizuka, Nanako Tamiya, Akihito Nakai, Keiya Fujimori, Tsugio Maeda, Hideaki Masuzaki, Hideaki Suzuki, Shigeru Ueda, Prevention Recurrence Committee, Japan Obstetric Compensation System for Cerebral Palsy

**Affiliations:** 1 Department of Obstetrics and Gynecology, St. Marianna University School of Medicine, Kanagawa, Japan; 2 Department of Public Health, The University of Tokyo, Tokyo, Japan; 3 President, The University of Miyazaki, Miyazaki, Japan; 4 Department of the Japan Obstetric Compensation System for Cerebral Palsy in Public Interest Incorporated Foundation, Japan Council for Quality Health Care, Tokyo, Japan; 5 Maternal and Perinatal Care Center, Oita Prefectural Hospital, Oita, Japan; 6 Department of Obstetrics and Gynecology, Mie University Graduate School of Medicine, Mie, Japan; 7 Department of Obstetrics and Gynecology, Showa University Northern Yokohama Hospital, Kanagawa, Japan; 8 Department of Health Services Research, Faculty of Medicine, University of Tsukuba, Ibaraki, Japan; 9 Department of Obstetrics and Gynecology, Nippon Medical School, Tokyo, Japan; 10 Department of Obstetrics and Gynecology, Fukushima Medical University, Fukushima, Japan; 11 Maeda Clinic, Incorporated association Anzu-kai, Shizuoka, Japan; 12 Department of Obstetrics and Gynecology, The University of Nagasaki, Nagasaki, Japan; Indiana University School of Medicine, UNITED STATES

## Abstract

**Objective:**

The aim of this study was to identify the relevant obstetric factors for cerebral palsy (CP) after 33 weeks’ gestation in Japan.

**Study design:**

This retrospective case cohort study (1:100 cases and controls) used a Japanese national CP registry. Obstetric characteristics and clinical course were compared between CP cases in the Japan Obstetric Compensation System for Cerebral Palsy database and controls in the perinatal database of the Japan Society of Obstetrics and Gynecology born as live singleton infants between 2009 and 2011 with a birth weight ≥ 2,000 g and gestation ≥ 33 weeks.

**Results:**

One hundred and seventy-five CP cases and 17,475 controls were assessed. Major relevant single factors for CP were placental abnormalities (31%), umbilical cord abnormalities (15%), maternal complications (10%), and neonatal complications (1%). A multivariate regression model demonstrated that obstetric variables associated with CP were acute delivery due to non-reassuring fetal status (relative risk [RR]: 37.182, 95% confidence interval [CI]: 20.028–69.032), uterine rupture (RR: 24.770, 95% CI: 6.006–102.160), placental abruption (RR: 20.891, 95% CI: 11.817–36.934), and preterm labor (RR: 3.153, 95% CI: 2.024–4.911), whereas protective factors were head presentation (RR: 0.199, 95% CI: 0.088–0.450) and elective cesarean section (RR: 0.236, 95% CI: 0.067–0.828).

**Conclusion:**

CP after 33 weeks’ gestation in the recently reported cases in Japan was strongly associated with acute delivery due to non-reassuring fetal status, uterine rupture, and placental abruption.

## Introduction

Cerebral palsy (CP) is a physical disability in children that is strongly associated with public health issues. Despite advances in obstetrics and perinatology, the incidence of CP is reported to be approximately 2 per 1,000 live births [1–3]. Although the risk of CP increases with preterm birth, it is not infrequent even in mature babies [3–5], with an incidence of 1.1 cases per 1,000 live births even at 40 weeks’ gestation [3].

The Japan Obstetric Compensation System for Cerebral Palsy (JOCSC) was launched in January 2009 because of a shortage of young obstetricians and regional gaps in obstetric care provision allegedly due to severe working environments and an increasing number of medical conflicts.

In the present report, obstetric characteristics, clinical course, and the relevant obstetric factors for CP after 33 weeks’ gestation in the JOCSC database (JOCSC-DB) were reviewed and compared with those of controls considered to be representative of the Japanese population in the perinatal database of the Japan Society of Obstetrics and Gynecology (JSOG-DB).

The purpose of the present study was to identify the relevant obstetric factors for CP in Japan. This is the first nationwide study to analyze and identify relevant obstetric factors for CP in Japan.

## Patients and Methods

A retrospective case-cohort study was conducted using a nationwide registry in Japan. Obstetric clinical characteristics and disease course were compared between the CP cases and controls.

Cases were infants with CP approved by a review of the Operating Organization of the JOCSC. The objectives of this JOCSC are to provide prompt no-fault compensation for children diagnosed with severe CP caused by trauma during labor and delivery and for their respective families, and to provide information that could help in the prevention, early resolution of disputes, and improvement of quality of obstetric health care. Compensation cases are reviewed by a review committee consisting of obstetricians, pediatricians, midwives, and lawyers according to the rules of the Operating Organization of the JOCSC. After being authorized as eligible to receive compensation by this review committee, the causes for CP are analyzed individually by the Causal Analysis Committee consisting of obstetricians, pediatricians, midwives, and lawyers. Once collected, these individual cases are analyzed from an epidemiological standpoint by the Recurrence Prevention Committee.

Those eligible for inclusion in the present study were infants born between January 2009 and December 2011 and those with a birth weight of ≥2,000 g, gestation of ≥33 weeks, and severe disability due to CP independent of congenital causes or factors during the neonatal period or later, with disability certified to be of first or second degree severity according to the grade of disability definitions in the Act for the Welfare of Persons with Physical Disabilities. The grade of disability range from Level 1 to Level 7, and Levels 1 and 2 correspond to severe locomotor disabilities. Specifically, Levels 1 and 2 indicate the bed-ridden state of a patient, and anticipated inability to walk in the future. According to the rules of the Operating Organization of the JOCSC, CP should be diagnosed by a physician with expertise in CP. Specifically, CP should be diagnosed by a pediatrician qualified for certification of patients with physical disabilities under the Act for the Welfare of Persons with Physical Disabilities or by a physician registered as a specialist in pediatric neurology with the Japanese Society of Child Neurology. Cases were excluded only if congenital anomalies such as bilateral extensive brain malformation, chromosomal aberration, gene anomaly, congenital metabolic abnormality, or other congenital anomaly were present, or neonatal factors such as meningitis and encephalitis were present and plausible causative factors determined by the Review Committee using head imaging data or other test data (medical records, MRI, CT, etc.), and if those factors were apparent principal causes of motor function disturbance.

To restrict cases and controls for demographics, only subjects born between January 2009 and December 2011 as live singleton infants with no major congenital anomalies at a general or university hospital or a perinatal center with a birth weight ≥ 2,000 g and gestation ≥ 33 weeks were enrolled. The analysis included cases wherein the final stages of labor occurred at these hospitals. Particularly, the analysis included cases of women transported from general physicians or private clinics to hospitals, in addition to the cases of women scheduled for delivery at hospitals. Thus, among the controls, all cases of stillbirth and neonatal death were excluded from the present study.

Controls in this retrospective case-cohort study were obtained from the perinatal database of the JSOG-DB, which is the nationwide registry in Japan established in 1974. For the JSOG-DB, attending physicians at 192 secondary and tertiary care centers of the Perinatal Research Network in Japan have collected yearly data for each pregnant woman through an off-line clinical database system using a common format. The data are stored by the Perinatal Committee of the JSOG following strict quality control of the information contained in the database [6]. As the JSOB-DB is the largest database, including about 10% of the total number of births and about 40% of cases resulting in perinatal death in Japan, it is available to clinical researchers in Japan and has been used in previous similar studies [7–10].

As a control subcohort, 17,500 newborns were randomly retrieved from the JSOG-DB, and 17,450 samples without data deficiencies were ultimately included as subjects in the present study. The following clinical variables associated with CP were analyzed in these subjects: (i) maternal age, height, weight, parity, and history of hypertension or *in vitro* fertilization, which were analyzed as maternal characteristics; (ii) pregnancy-induced hypertension (PIH), diabetes mellitus (DM), gestational DM (GDM), preterm labor, premature rupture of membrane (PROM), intra-amniotic infection, placental abruption, polyhydramnios, and oligohydramnios, which were analyzed as variables during pregnancy; and (iii) maternal body weight at delivery, weight gain during pregnancy, oxytocin and prostaglandin augmentation, head presentation of the fetus, uterine rupture, instrumental delivery, acute delivery due to non-reassuring fetal status (NRFS), and both elective and emergency cesarean section, which were analyzed as variables at delivery. Finally, number of gestational weeks, birth weight, male sex, Apgar score, and umbilical artery pH were compared as neonatal variables.

### Definitions

#### Cerebral palsy

CP is defined as the disturbance of motor function or posture of infants that is permanent or variable. The disorder is based on a non-progressive cerebral lesion that develops anytime between conception to the neonatal period (within 4 weeks after birth). However, this definition excludes motor retardation that is either transient or normalizes in the future.

#### PIH

The definition of PIH included preeclampsia (PE) and gestational hypertension (GH). GH was defined as the onset of hypertension after 20 weeks’ gestation (defined as systolic blood pressure ≥ 140 mmHg or diastolic blood pressure ≥ 90 mmHg) on at least two occasions 4 h apart. PE was defined as GH with proteinuria (0.3 g in a 24-h urine specimen or a protein to creatinine ratio of >0.30).

#### Intra-amniotic infection

Intra-amniotic infection was defined as an observed infection of the amniotic fluid, membranes, placenta, or decidua, based on overt clinical signs such as PROM, fever, uterine tenderness, maternal and fetal tachycardia, and foul amniotic fluid and/or pathological findings such as chorioamnionitis and/or funisitis or amniotic fluid with a positive bacterial culture.

#### Acute delivery due to NRFS

Acute delivery included emergency cesarean section, vacuum extraction, and forceps delivery due to a non-reassuring cardiotocogram.

### Statistical analysis

A two-side p-value of 0.05 was used to define statistical significance. All analyses were conducted using Stata version 13.0 (STATA Corporation, College Station, TX, USA). Continuous variables are reported as mean ± standard deviation and were compared using the Student *t*-test. Integer variables are reported as median and range, and were compared using the Mann—Whitney *U*-test. Categorical variables are reported as frequencies and were compared using the chi-square test. Relationships among clinical variables were evaluated using univariate and multivariate logistic regression with CP as the independent variable. Results are expressed by relative risk (RR) and 95% confidence interval (CI). Considering statistical collinearity among maternal weight, height, and BMI at pregnancy and data deficiency in Apgar score and umbilical artery pH among CP cases; independent variables including maternal age, height, and weight at pregnancy, multiparous birth, IVF, PIH, DM/GDM, preterm labor, PROM, placental abruption, polyhydramnios, oligohydramnios, weight gain during pregnancy, oxytocin and prostaglandin augmentation, uterine rupture, head presentation, acute delivery due to NRFS, instrumental delivery, elective cesarean section, emergency cesarean section, gestational weeks at delivery, neonatal birth weight, and male-sex infant were compulsorily entered into a multivariate model. The goodness of fit of the multivariate model was evaluated using the area under the operating characteristic (AUC) curve and the Hosmer-Lemeshow test. Collinearity among all variables in the final model was evaluated using variance inflation factor (VIF) with a cut-off value of 2.5.

### Ethics

The study protocol was approved by the Institutional Review Boards (IRBs) of the JOCSC. Written informed consent was not obtained from patients. However, patients were provided with a supplemental file that contained the announcement of implementation of a “case-control study for cerebral palsy and prevention of its recurrence”. Although the analysis was retrospective, the data for the anonymized JOCSC-DB and the JSOG-DB collected in a normal clinical setting ensured that the confidentiality of the patients involved was protected. All patient records/information was anonymized and de-identified prior to analysis and no personal data were necessary for the present study.

## Results

**Fig 1:**
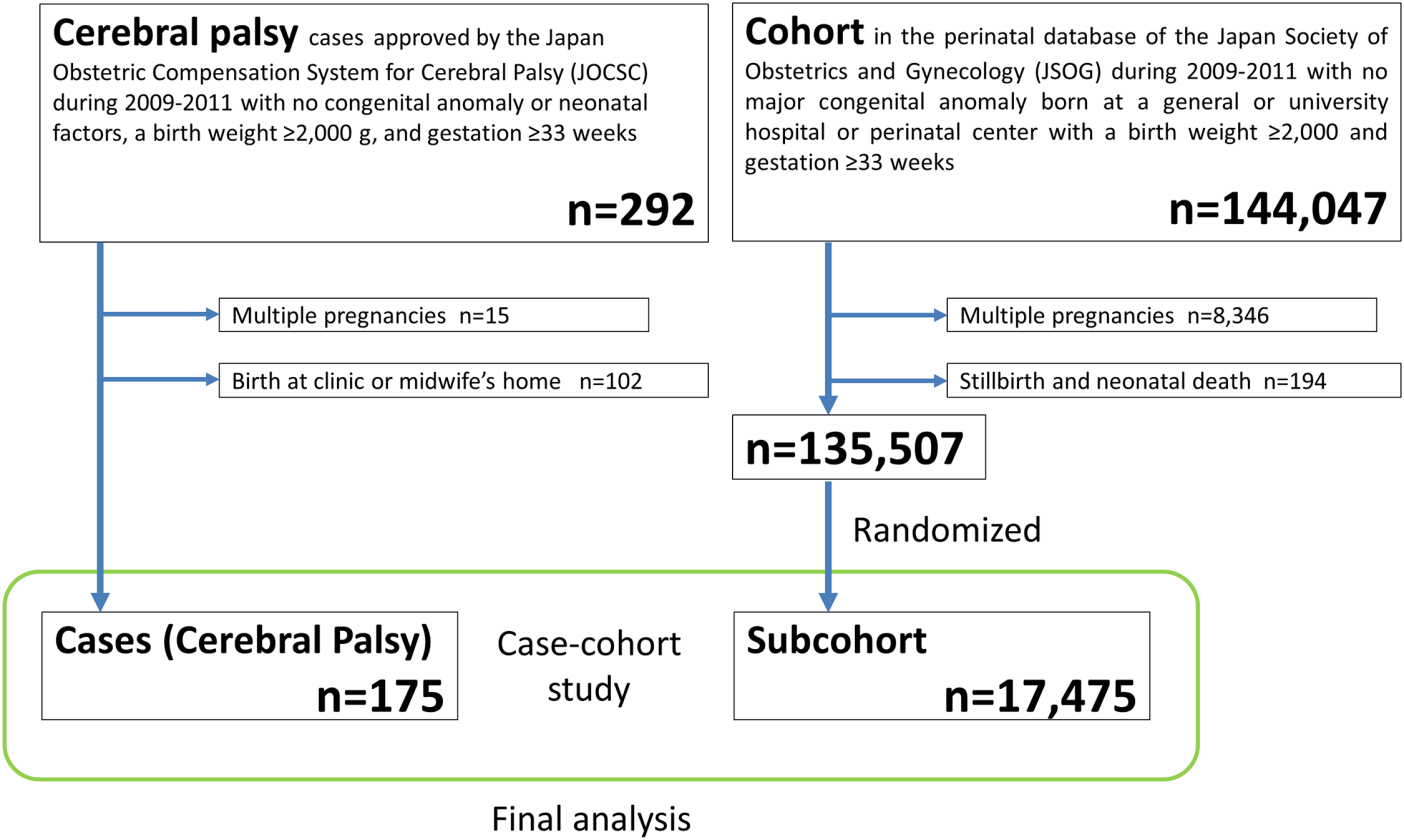
Study flow diagram.

The data of 292 infants with CP obtained from the JOCSC-DB and those of 144,047 infants used to represent the Japanese population from the JSOG-DB restricted for birth weight (≥2,000 g) and pregnancy duration (≥33 weeks) were available for this analysis. The study flow diagram is shown in Fig 1. Fifteen infants with CP and 8,346 infants of multiple births from the general population were excluded from the present study. Moreover, 102 infants with CP born at private clinics or at midwives’ houses were excluded from the CP group because the study populations were limited to patients born at a tertiary hospital. In addition, 194 cases of stillbirths and neonatal deaths were excluded from the general population. After exclusion of the above infants, a total of 135,507 infants were available for analysis as the population control sample pool; of them, 17,475 control infants were randomly selected using computer software. Overall, 175 cases and 17,475 controls were enrolled as the subjects of the present study and analyzed as a 1:100 case-cohort study.

The characteristics of the cases and the major relevant obstetric factors for CP analyzed by the JOCSC Causal Analysis Committee and classified by the Recurrence and Prevention Committee in the 175 CP cases are shown in Table 1. Relevant single factors considered were placental abnormalities (31%), umbilical cord abnormalities (15%), maternal complications (10%), and neonatal complications (1%). About 26% of CP cases were unexplained.

**Table 1:** Characteristics of cases—major relevant obstetric factors for cerebral palsy reviewed by the Operating Organization (n = 175).

**Single factor**	**99 (57%)**	
Placental abnormalities		55 (31%)
Placental abruption		50
Bleeding of placenta previa		1
Feto-maternal blood transfusion		4
Umbilical cord abnormalities		26 (15%)
Umbilical cord prolapse		9
Other cord abnormalities		17
Maternal complications		18 (10%)
Uterine rupture		7
Intraamniotic infection		3
Sepsis		1
Herpetic meningoencephalitis		1
Maternal cardio-pulmonary arrest		2
Amniotic fluid embolism		1
PIH		2
Uncontrolled DM		1
Neonatal complications		2 (1%)
Blood type incompatibility		1
Intraventricular hemorrhage		1
**Multifactorial**	**29 (17%)**	
Placental abruption		
+ cord abnormality		1
+ FGR		1
+ uterine rupture		1
+ intraamniotic infection		1
+ maternal shock		1
Cord abnormality		
+ intraamniotic infection		7
+ intraventricular hemorrhage		1
+ shoulder dystocia		1
FGR		
+ intraamniotic infection		2
+ PIH		2
+ velamentous insertion		1
Uncontrolled DM		
+ intraventricular hemorrhage		1
Other causes		9
**Unexplained**	**45 (26%)**	

PIH; pregnancy induced hypertension, DM; diabetes mellitus, FGR; fetal growth reastriction. Other cord abnormalities included velamentous/marginal cord insertion, vasa previa, hyper coiled cord, cord entanglement, constriction of umbilical cord, and long umbilical cord. Other causes included meconium aspiration syndrome, hyperglycemia, maternal shock, and hypertonic uterine contraction.

Obstetric characteristics, clinical course, and the relevant obstetric factors identified in CP cases and the subcohort are shown in Table 2. Variables up to the time of delivery that had a high crude RR were placental abruption, acute delivery due to NRFS, uterine rupture, and emergency cesarean section. In our series, 8 cases (4.6%) of uterine rupture were observed. Half of these cases occurred suddenly outside the hospital, whereas the other half occurred during labor in a hospital setting. Six cases (75%) had risk factors such as a history of cesarean section and bicornuate uterus. One case was subjected to vacuum extraction with a uterine fundal pressure maneuver. The frequencies of intra-amniotic infection, velamentous cord insertion, marginal cord insertion, and umbilical cord prolapse were not available for controls because JOSG-DB did not collect these data.

**Table 2:** Obstetric characteristics, clinical course, and relevant obstetric factors in cases with cerebral palsy and subcohort.

Variables	Cases	Control subcohort	Crude	95% Confidence
	(n = 175)	(n = 17,475)	relative risk	interval
Maternal				
Age	31.3 ± 5.7	31.9 ± 5.3	0.981	0.954–1.001
Height (cm)	157.7 ± 5.6	158.3 ± 5.5	0.98	0.954–1.007
Weight (kg)	54.8 ± 11.4	53.2 ± 9.9	1.014	1.001–1.027
BMI (kg/m2)	22.0 ± 4.8	21.2 ± 3.7	1.047	1.015–1.081
Parity (median, range)	0 (0–5)	0 (0–9)	0.955	0.797–1.145
Hypertension	0.0% (0)	0.8% (143)	n/a	
IVF	5.1% (9)	4.3% (758)	1.196	0.609–2.348
During pregnancy				
PIH	8.0% (14)	4.0% (704)	2.072	1.194–3.595
DM/GDM	2.9% (5)	4.0% (694)	0.711	0.291–1.736
Preterm labor	32.0% (56)	12.4% (2,159)	3.338	2.422–4.601
PROM	15.4% (27)	11.6% (2,019)	1.397	0.924–2.110
Intraamniotic infection	18.3% (32)	n/a	n/a	
Placental abruption	32.6% (57)	0.6% (104)	80.683	55.715–116.841
Velamentous insertion/vasa previa	4.6% (8)	n/a	n/a	
Marginal insertion	12.6% (22)	n/a	n/a	
Polyhydramnios	1.1% (2)	0.7% (120)	1.712	0.420–6.980
Oligohydramnios	2.9% (5)	1.6% (288)	1.798	0.733–4.409
At delivery				
Weight (kg)	64.3 ± 11.1	62.9 ± 9.7	1.014	1.000–1.028
Weight gain (kg)	9.6 ± 4.2	9.7 ± 4.4	0.993	0.959–1.027
Oxytocin augmentation	24.6% (43)	23.7% (4,144)	1.048	0.741–1.481
Prostaglandin augmentation	6.9% (12)	6.1% (1,065)	1.134	0.629–2.045
Uterine rupture	4.6% (8)	0.1% (17)	49.195	20.942–115.564
Head presentation	91.4% (160)	95.4% (16,672)	0.514	0.301–0.876
TV breech presentation	1.7% (3)	0.09% (16)	16.185	5.669–46.207
Umbilical cord prolapse	5.1% (9)	n/a	n/a	
Acute delivery due to NRFS	68.6% (120)	3.2% (566)	65.181	46.862–90.661
Mode of delivery				
Normal spontaneous	21.1% (37)	65.8% (11,493)	reference	
Instrumental	13.7% (24)	7.1% (1,238)	6.021	3.591–10.099
Elective CS	1.7% (3)	16.6% (2,895)	0.322	0.099–1.045
Emergency CS	63.4% (111)	10.6% (1,849)	18.647	12.812–27.140
Neonate				
Gestational weeks	38.1 ± 2.0	38.6 ± 1.5	0.817	0.747–0.892
Birth weight (g)	2,846 ± 457	2,969 ± 399	0.924	0.889–0.960
(SD)	-0.14 ± 1.04	-0.02 ± 0.98	0.879	0.752–1.027
Small for gestational age	11.4% (20)	5.5% (958)	2.225	1.390–3.560
Male	51.4% (90)	50.9% (8,902)	1.02	0.757–1.374
Apgar Score				
1 min.				
> = 7	6.3% (11)	96.7% (16890)	reference	
4–7	11.4% (20)	2.3% (410)	74.9	35.657–157.335
<4	56.6% (99)	1.0% (168)	904.821	476.540–1718.013
Unknown	25.7% (45)	0.04% (7)		
5 min.				
> = 7	10.9% (19)	99.2% (17338)	reference	
4–7	33.1% (58)	0.6% (101)	524.024	301.168–911.704
<4	36.6% (64)	0.2% (28)	2085.772	1108.473–3924.719
Unknown	19.4% (34)	0.05% (8)		
Umbilical artery pH				
> = 7.2	13.7% (24)	92.0% (16082)	reference	
7.0–7.2	16.0% (28)	7.7% (1338)	14.023	8.106–24.258
6.8–7.0	16.0% (28)	0.3% (46)	407.877	220.018–756.136
<6.8	21.1% (37)	0.05% (9)	2754.788	1199.741–6325.415
Unknown	33.1% (58)	0% (0)		

Data indicate mean ± standard deviation, median (range) or frequency (n). n/a; not available, IVF; in vitro Fertilization, PIH; pregnancy induced hypertension, DM; diabetes mellitus, GDM; gestational diabetes mellitus, PROM; premature rupture of membrane, TV; trans vaginal delivery, NRFS; non-reassuring fetal status, CS; cesarean section, SD; standard deviation.

The multivariate regression model including all the variables is shown in Table 3. Obstetric variables strongly associated with CP were acute delivery due to NRFS (adjusted RR [aRR]: 37.182, 95% CI: 20.028–69.032), uterine rupture (aRR: 24.770, 95% CI: 6.0065.726–102.160), and placental abruption (aRR: 20.891, 95% CI: 11.817–36.934). On the other hand, the obstetric variables associated with reduced CP were head presentation (aRR: 0.199, 95% CI: 0.088–0.450) and elective cesarean section (aRR: 0.236, 95% CI: 0.067–0.828).

**Table 3:** Results of the multiple logistic regression analysis.

Variables	Adjusted relative risk	95% Confidence interval
Maternal weight at pregnancy (kg)	1.035	1.017–1.054
Preterm labor	3.153	2.024–4.911
Placental abruption	20.891	11.817–36.934
Oxytocin augmentation	1.612	1.034–2.513
Uterine rupture	24.77	6.006–102.160
Head presentation	0.199	0.088–0.450
Acute delivery due to NRFS	37.182	20.028–69.032
Elective Cesarean section	0.236	0.067–0.828

Covariates: maternal age, height, multiparous, IVF, PIH, DM/GDM, PROM, polyhydramnios, oligohydramnios, weight gain during pregnancy, prostaglandin augmentation, instrumental delivery, emergency caesarean section, gestational week at delivery, small for gestational age and male infant; N = 17,633; Hosmer-Lemeshow test (degrees of freedom: 8) P = 0.496; Area under operating characteristic curve: 0.913.

The AUC of the final model of 0.91 indicated a high predictive ability. The Hosmer-Lemeshow test for the final model was non-significant (chi-square: 7.39, degrees of freedom: 8, p = 0.496), indicating that the model performed equally well for all variables. The VIF for all variables in the final model was <2.5, indicating that all variables were orthogonal to each other.

## Discussion

In summary, we found a strong association between CP and acute delivery due to NRFS among infants with a birth weight of ≥2,000 g and gestation length of ≥33 weeks. In fact, umbilical artery pH was <7.0 in 37% of CP cases but only in 0.35% of controls. Multiple logistic regression analysis identified eight obstetrical conditions that independently affected the risk of developing of CP; of them, three factors, including placental abruption, uterine rupture, and acute delivery due to NRFS, had extremely large effects.

Intrauterine growth restriction induced by antenatal conditions and resulting in low birth weight for gestational age and preterm delivery are the main risk factors of CP [4, 11–14]. However, in CP infants born almost matured at near term such in the present subjects, adverse conditions involving acute utero-placental under-perfusion including placental abruption and uterine rupture, and required acute delivery due to NRFS were more associated with CP rather than chronically progressive adverse condition such as fetal growth restriction and hypertensive disorder. Previous meta-analysis demonstrated that newborns delivered by emergency Cesarean section at term were more likely to have CP (1.6; 95%CI 1.05–2.44) [15]. It is considered that this result showed risk factors for CP in association with emergency Cesarean section, with the likely confounding that acute or chronic fetal compromise would often precipitate this intervention [15–17].

A high aRR with acute delivery due to NRFS, placental abruption, and umbilical artery pH were also consistent with speculations aboutacidemia, as these factors are known to associate with an increased risk of hypoxemia and academia, resulting in CP. Acute deliveries due to NRFS were thought to involve many umbilical cord complications. Although data relative to frequencies of umbilical cord abnormalities were not available for controls in the present study, umbilical cord prolapse was more frequently observed in our cases with CP (5.1%) than in previous reports (0.12–0.62%)[18–21]. An abnormally positioned fetus is less likely to be engaged in the maternal pelvis, non-vertex presentation is associated with umbilical cord prolapse [22, 23]. Our result that head presentation was protective is consistent with previous studies shown increased risk of CP in term infants born in breech presentation [4, 24, 25]. Velamentous cord insertion was also more frequently observed in our cases with CP (4.6%) than in previous reports ranging from 0.5% to 1.69% of singleton pregnancies [26, 27]. Therefore, strong associations between CP and pathological conditions of the placenta and umbilical cord that require acute delivery are suspected.

A previous study supported the usefulness of fetal heart rate monitoring in the detection of fetal acidemia in low-risk pregnancies and concluded that under continuous monitoring, CP caused by intrapartum asphyxia was restricted to unavoidable hypoxic accidents [28]. The exposure duration to adverse conditions such as hypoxemia and acidemia also increases the risk of adverse outcomes and, hence, could indicate where clinical efforts might result in improvements by either reducing their incidence or shortening the delivery time [29]. Obstetric system improvements consisting of suitable emergency obstetric staffing, education, and training in hospitals might be required in order to reduce CPs caused due to acute intrapartum accident. In particular, iatrogenic accidents during labor, such as umbilical cord prolapse due to amniotomy without confirmation of the descending cord using ultrasound [30], and uterine rupture associated with uterine fundal pressure maneuver [31], should be reduced.

On the other hand, fear of CP litigation is a major influence on the defensive decision-making of the obstetrician to perform a Cesarean section [32, 33]. Nevertheless, the pathogenesis of CP has been attributed to antenatal as well as intrapartum factors [34, 35]. A review concluded that 70–80% of CP cases were due to prenatal factors and that birth asphyxia played a relatively minor role (<10%) [35]. Reportedly >90% of term and near-term singleton infants with CP had experienced no recognized potentially asphyxiating birth event [36, 37]. In the present study, pre-admission central nervous system injury was suspected in some cases because there was a persistent loss of heart rate variability without deceleration of the tracing at the time of admission. In fact, no relevant obstetric factors were identified in 26% of CP cases, and the umbilical artery pH was >7.0 in approximately 30% of CP cases. These results might indicate that CP is not preventable, even if an early emergency or elective cesarean section is performed.

Low blood flow in the placenta, brain, or other organs and inflammation confer a clotting predisposition and increased thrombosis risk [37]. When such events occur relatively early in pregnancy, they can cause brain lesions; later in pregnancy, they can cause cerebral infarction in a vascular territory [37]. In neurologically impaired term infants, umbilical cord abnormalities such as abnormal cord insertion and umbilical cord entanglement are significantly increased in placentas with fetal thrombotic vasculopathy [38]. Even placenta abruption has important antecedents including placental inflammatory and vascular disorders, although the most catastrophic event occurs at birth [39]. Therefore, it is considered that no protective effect of elective Cesarean section in term infant for CP was plausible results [4, 15].

## Limitations and Strengths

As this is the first large nationwide study in Japan to analyze obstetrically relevant factors for CP after 33 weeks’ gestation, we believe that the results reported herein will provide useful information and thus reduce the incidence of CP cases associated with acidemia near the time of delivery and with obstetric management.

Limitations of the present study include several biases of the study design. Because there is no registration or record system, archived obstetric and long-term pediatric characteristics, different subjects from two databases, CP cases from the JOCSC-DB and controls from the JSOG-DB were compared. Since characteristics were restricted between the two groups, selection bias was practically undetectable. A subcohort sample to analyze the two groups was collected, but it may have contained a few CP cases. It is likely that the JOCSC-DB may also be subject to information bias, because the JSOG-DB simply contains records from a registration software whereby data are entered by caregivers from obstetric institutions after delivery; whereas, the JOCSC-DB was designed by expert staff according to precise criteria, and data input from medical records required for evaluation of CP cases to qualify for compensation eligibility.

Furthermore, in the present study, the multivariate regression analysis showed that elective Cesarean section was protective for occurrence of CP, in contrast to the result of univariate analysis, while in the previous studies no protective effect for CP was shown for elective Cesarean section in term infant [4, 15]. This discrepancy might be attributable to the lack of available data related to the frequencies of placental and umbilical cord abnormalities, including velamentous/marginal cord insertion, vasa previa, umbilical cord prolapse and intra-amniotic infection, for multivariate regression analysis in JOSG-DB. Therefore, it is difficult to determine whether elective cesarean section has a favorable effect with regard to CP reduction. The collection of further detailed data associated with antenatal causes of CP is needed in JOSG-DB.

## Conclusion

In the present study, CP was strongly associated with acute delivery due to NRFS, uterine rupture, and placental abruption. These acute hypoxic academic situations were indicated by a low Apgar score and low umbilical artery blood pH. Although cases of CP with chronic, progressive antenatal causes are unavoidable, we believe that improvements made to obstetric management during labor can at least reduce the incidence of CP cases resulting from delivery-related complications.

## Supporting Information

S1 File Announcement of implementation of a “case-control study for cerebral palsy and prevention of its recurrence” for patients. (DOCX)
