# Supplementary material for: Relevant Obstetric Factors for Cerebral Palsy: From the Nationwide Obstetric Compensation System in Japan
Source: PLoS One. 2016 Jan 28;11(1):e0148122. doi: 10.1371/journal.pone.0148122 (PMC4731141; doi:10.1371/journal.pone.0148122)
Supplement: S1 File — (DOCX) [file pone.0148122.s001.docx]

**Japan Obstetric Compensation System for Expectant and Nursing Mothers:**

**Implementation of a “case-control study for cerebral palsy and prevention of its recurrence”**

The Operating Division of the Obstetric Compensation System of the Japan Council for Quality Health Care (JCQHC) will conduct a case-control study^Note)^ with the aim of identifying causes of cerebral palsy (CP) and measures to prevent CP relapse by comparing affected cases compensated according to the Japan Obstetric Compensation System for Cerebral Palsy (JOCSC) with non-CP cases in the perinatal registry database of the Japan Society of Obstetrics and Gynecology (JSOG). The main study goals are to prevent the recurrence of similar cases and enhance the quality of obstetric care in the Working Group for Prevention of Relapse of Cerebral Palsy, which is composed of members of the Recurrence and Prevention Committee, obstetricians recommended by the JSOG and the Japan Association of Obstetricians and Gynecologists, academic experts, and so on.

The main study material will be the Causal Analysis Report, which was prepared based on medical care information submitted by the delivery institutions where pregnant women gave birth to their children when applying for this compensation system or at the time of causal analysis, and the perinatal registry database of the JSOG. This database was provided by the JSOG after filing the application to the Information Management Committee of the JSOG. Both the report and the database contain information on cases delivered from 2009 to 2011.

Since the medical care information submitted by the delivery institutions is stored under strict conditions by this Council after making all personal information anonymous at the point of initiating the analysis for this study, any personal information pertaining to participant privacy will never be divulged to outside persons. Also, there will be no burdens on study participants. As a prospective participant, if you tell us that you cannot cooperate in this study, we will exclude you from the analysis. However, please note that we cannot exclude you from the analysis after its initiation because the information will be stored in a form that does not allow individuals to be identified.

The study results will be used as a part of our efforts within this Council to prevent the recurrence of CP and will be published in the “Report on Recurrence and Prevention of CP for the Obstetric Compensation System”, as a research paper in an academic journal, the website of the Compensation System, and by other appropriate means.

The implementation of this study has been approved by the Ethics Committee of this Council.

Please contact us if you have any concerns and questions.

Note) A study that clarifies the causes, background factors, etc., of cerebral palsy by statistically comparing and analyzing CP cases compensated according to this Compensation System with non-CP cases.

August 13, 2014

Japan Council for Quality Health Care (JCQHC)
